# Impaired fornix–hippocampus integrity is linked to peripheral glutathione peroxidase in early psychosis

**DOI:** 10.1038/tp.2016.117

**Published:** 2016-07-26

**Authors:** P S Baumann, A Griffa, M Fournier, P Golay, C Ferrari, L Alameda, M Cuenod, J-P Thiran, P Hagmann, K Q Do, P Conus

**Affiliations:** 1 Department of Psychiatry, Unit for Research in Schizophrenia, Center for Psychiatric Neuroscience, Centre Hospitalier Universitaire Vaudois, Lausanne University Hospital (CHUV), University of Lausanne, Lausanne, Switzerland; 2 Department of Psychiatry, Service of General Psychiatry, Centre Hospitalier Universitaire Vaudois, Lausanne University Hospital (CHUV), Lausanne, Switzerland; 3 Signal Processing Laboratory (LTS5), Ecole Polytechnique Fédérale de Lausanne, Lausanne, Switzerland; 4 Department of Radiology, Lausanne University Hospital (CHUV), University of Lausanne, Lausanne, Switzerland; 5 Service of Community Psychiatry, Department of Psychiatry, Lausanne University Hospital (CHUV), Lausanne, Switzerland

## Abstract

Several lines of evidence implicate the fornix–hippocampus circuit in schizophrenia. In early-phase psychosis, this circuit has not been extensively investigated and the underlying mechanisms affecting the circuit are unknown. The hippocampus and fornix are vulnerable to oxidative stress at peripuberty in a glutathione (GSH)-deficient animal model. The purposes of the current study were to assess the integrity of the fornix–hippocampus circuit in early-psychosis patients (EP), and to study its relationship with peripheral redox markers. Diffusion spectrum imaging and T1-weighted magnetic resonance imaging (MRI) were used to assess the fornix and hippocampus in 42 EP patients compared with 42 gender- and age-matched healthy controls. Generalized fractional anisotropy (gFA) and volumetric properties were used to measure fornix and hippocampal integrity, respectively. Correlation analysis was used to quantify the relationship of gFA in the fornix and hippocampal volume, with blood GSH levels and glutathione peroxidase (GPx) activity. Patients compared with controls exhibited lower gFA in the fornix as well as smaller volume in the hippocampus. In EP, but not in controls, smaller hippocampal volume was associated with high GPx activity. Disruption of the fornix–hippocampus circuit is already present in the early stages of psychosis. Higher blood GPx activity is associated with smaller hippocampal volume, which may support a role of oxidative stress in disease mechanisms.

## Introduction

The fornix–hippocampus circuit^1^ is part of the classic Papez circuit,^2^ which has a crucial role in spatial memory, memory retrieval and verbal memory,^3, 4^ functions that are affected in schizophrenia.^5^ Studies investigating the hippocampus in schizophrenia highlighted volume loss,^6, 7, 8, 9^ altered diffusion properties^10, 11, 12^ and hypermetabolism^13, 14^ at the neuroimaging level and decrease in parvalbumin-immunoreactive γ-aminobutyric acid interneurons at the microscopic level.^14, 15, 16^

Given its direct anatomical link with the hippocampus, diffusion magnetic resonance imaging (MRI) studies also focused on the fornix, a fine compact, arch-shaped white matter bundle connecting the hippocampus to the hypothalamus, and various other cortical and subcortical structures including mammillary bodies.^17^ These studies consistently showed a decreased fractional anisotropy (FA) in the fornix in chronic schizophrenia.^4, 18, 19, 20, 21, 22^ Hippocampal volume (HV) correlates with the mean diffusivity in the fornix in patients only, indicating important structural relationship between these structures in disease.^22^ Interestingly, this tight relationship between the fornix and hippocampus is also present in Alzheimer’s disease,^1, 23^ hippocampal sclerosis in mesial temporal lobe epilepsy^24, 25, 26^ and multiple sclerosis.^27^ Neuropathological characterization of the fornix in schizophrenia showed no differences in fiber number but higher fiber density in the fornix in male schizophrenia patients compared with controls.^28^

The imbalance between oxidant and antioxidant systems is emerging as an important pathophysiological hub in schizophrenia^29, 30, 31, 32, 33^ and may contribute to the microstructural alteration of the fornix–hippocampus circuit. Dysregulation of glutathione (GSH) synthesis, the major non-protein cellular antioxidant, is critically involved in a subgroup of schizophrenia patients.^30, 34^ The effect of redox dysregulation on the brain has been studied in transgenic mice with a deletion of the modifier subunit of glutamate cysteine ligase (that is, *Gclm*-KO mice), which leads to a 70% decrease in GSH brain levels and several schizophrenia-related phenotypes.^30, 35^ Recently, a longitudinal 14-Tesla diffusion tensor imaging study in *Gclm*-KO mice showed a decrease in FA in the fornix.^36^ Diffusion tensor imaging parameters were altered in peripubertal knockout mice and remained altered in adulthood. Electrophysiological recordings in the same model showed a significant decrease in conduction velocity in the fimbria–fornix fibers, providing a potential functional basis of FA alterations. This study underlines the high vulnerability of the fornix to oxidative stress induced by GSH deficit. Our recent report also supports the critical role of GSH and redox regulation in the myelination processes and white matter maturation.^37, 38^ At the cellular level, research in the same model showed that hippocampus fast-spiking parvalbumin γ-aminobutyric acid interneurons and their synchronization are also impaired, all features known to be affected in schizophrenia.^39^ In summary, preclinical research indicates that redox imbalance affects the fornix and hippocampus, with relevance to schizophrenia. The critical vulnerability of the fornices and hippocampi to oxidative stress during development fueled new hypotheses. Fornix alterations around puberty in *Gclm-KO* mice would predict potential white matter anomalies in the early phase of psychosis as well as a link with GSH/redox systems. Capturing the peripheral redox balance is not straightforward, and studies on the various antioxidant systems in the peripheral tissue of schizophrenia patients showed large discrepancies between studies,^31, 40, 41^ which may be due to different stages of disease (acute versus chronic or active versus remission phase), differences in analytical methodologies, testing materials (blood cells versus plasma or serum), exposure to medication, lifestyle (for example, smoking) or dietary intake.^31, 41^ Glutathione peroxidases (GPx) are an important selenium-dependent antioxidant enzyme family that eliminates hydrogen and lipid peroxides by oxidizing GSH and are thus an effective protection against cellular injuries. The oxidized GSH is then reduced back by the GSH reductase. Recent studies showed reduced FA in the fornix in first-episode psychosis patients;^42, 43^ however, its relationship to HV and peripheral redox markers in the early phase of psychosis has, to the best of our knowledge, never been tested before.

From a reverse translational train of thought, from models to patients, we thus aim to test (1) the presence of white matter alterations in the fornix and its relationship with hippocampus integrity in early-psychosis (EP) patients thanks to diffusion spectrum imaging (DSI) and volumetry. (2) The correlation between structural integrity in the fornix–hippocampus circuit and peripheral GPx activity and GSH levels.

## Materials and methods

### Subjects

EP patients, having met threshold criteria for psychosis, as defined by the ‘Psychosis threshold’ subscale of the Comprehensive Assessment of At Risk Mental States^44^ were recruited from the TIPP Program (Treatment and Early Intervention in Psychosis Program, University Hospital, Lausanne, Switzerland).^45^ This EP program offers 3 years of treatment to patients aged 18–35 years. Diagnoses were assessed according to the Diagnostic and Statistical Manual of Mental Disorders criteria. Healthy controls, recruited from similar geographic and sociodemographic areas through advertisement, were assessed by the Diagnostic Interview for Genetic Studies^46^ and matched on gender, age and handedness. Major mood, psychotic or substance-use disorder as well as having a first-degree relative with a psychotic disorder were exclusion criteria for controls. Neurological disorders and severe head trauma were exclusion criteria for all subjects. In both patients and controls, cigarette smoking and cannabis status were recorded (user or non-user) as well as weight and height in order to calculate body mass index (BMI; kg/m^2^). For patients, daily cigarette consumptions and cannabis use assessed with the Case Manager Rating Scale (CMRS; adapted from Drake *et al.*;^47^ 1=non, 2=mild, 3=moderate, 4=severe and 5=extremely severe) were also available. All assessments (MRI, blood and clinical) were performed at the same time point. Symptomatic severity was assessed with the Positive and Negative Syndrome Scale (PANSS) administered by a trained psychologist. Antipsychotic doses at the time of the study were converted to chlorpromazine equivalents (CPZ equivalents in mg)^48^ for each patient. Informed written consent in accordance with our institutional guidelines (protocol approved by the Ethic Committee of Lausanne University) was obtained for all the subjects.

### MRI acquisition and analysis

#### MRI acquisition

MRI sessions were performed on a 3-Tesla scanner (Magnetom TrioTim, Siemens Medical Solutions, Erlangen, Germany) equipped with a 32-channel head coil. Each scanning session included a magnetization-prepared rapid acquisition gradient echo (MPRAGE) T1-weighted sequence with 1-mm in-plane resolution and 1.2-mm slice thickness, covering 240 × 257 × 160 voxels. The repetition (TR), echo (TE) and inversion (TI) times were, respectively, 2300, 2.98 and 900 ms. The DSI sequence included 128 diffusion-weighted images with a maximum *b-*value of 8000 s mm^−^^2^ and one b0 reference image. The acquisition volume was made of 96 × 96 × 34 voxels with 2.2 × 2.2 × 3 mm resolution. TR and TE were, respectively, 6800 and 144 ms.

#### MRI analysis

All images were visually inspected for artifacts or structural abnormalities. Diffusion and T1-weighted MRI data were processed using the Connectome Mapping Toolkit (http://www.cmtk.org/).^49, 50, 51^ MPRAGE volumes were segmented into white matter, gray matter and cerebrospinal fluid compartments, and were linearly registered to the b0 volume. Gray matter and subcortical structures (notably the hippocampus) were segmented using the FreeSurfer software (version 1.313.2.6; https://surfer.nmr.mgh.harvard.edu/).^52^ DSI data were reconstructed according to Weeden *et al.,*^53^ allowing to estimate multiple diffusion directions per voxel. Deterministic streamline tractography^54^ was performed on DSI-reconstructed data.

Generalized FA (gFA), calculated as described by Tuch *et al.*,^55^ is similar to the concept of FA and describes the local degree of anisotropy of a diffusion process while compensating for multiple orientations within single voxels.

**Figure 1:**
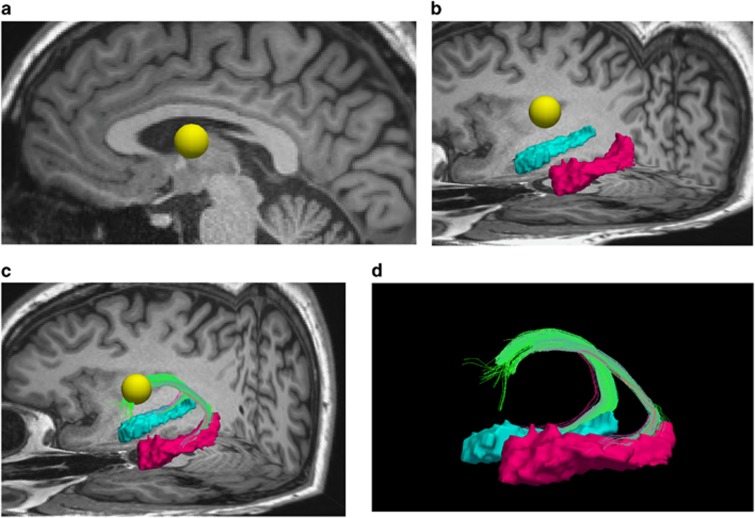
Fornix segmentation method. (**a**) Placement of a sphere (in yellow) in the body of the fornix on T1 scan (sagittal view). (**b**) Selection of left (pink) and right (blue) hippocampi. (**c**) The fornix was defined as fibers connecting the sphere and the left and right hippocampi. (**d**) Lateral view of the fornix bundle (in green). Posterior part of the brain is on the right side of each figure.

The fornix segmentation method was developed for the current study using the Trackvis software (http://trackvis.org).^56^ The only manual procedure was the placement of a sphere of diameter 10 mm in the body of the fornix, which was performed on the MPRAGE by the investigator (PSB), blind to diagnosis. Bilateral hippocampi were selected according to FreeSurfer segmentation.^52^ Then, fiber tracts connecting the left and right hippocampi to the sphere were selected. Length thresholding (streamlines longer than 80 mm were discarded) allowed an exquisite delineation of the fornix (columns, body and crura; Figure 1). All parameters (sphere size and fiber length thresholding) were set identically for all subjects. Then, average gFA was extracted along the left and right fornix.

HV was obtained from the FreeSurfer software segmentation. HV of each individual subject was corrected for intracranial volume (ICV) by computing the ratio (HV in mm^3^/ICV in mm^3^).

### Peripheral GPx activity and GSH levels

Blood was collected by venipuncture between 0700 and 0830 hours under restricted activity conditions and fasting from the previous midnight. Blood cells were prepared as in Gysin *et al.*^57^ Vacutainer tubes coated with Li-heparinate (Becton Dickinson, Franklin Lakes, NJ, USA), previously placed on ice, were used to collect 18–20 ml blood. An aliquot of whole blood was sampled and frozen at −80 °C until analysis of GSH content. The rest of the blood was centrifuged at 3000* g*, 5 min, 4 °C; the pellet, corresponding to blood cells, was washed two times with 0.9% NaCl and was frozen at −80 °C until analysis. All manipulations were performed rapidly with cooling to avoid artefactual oxidation of thiol compounds.

The activity of GPx was determined according to Günzler *et al.*^58^ In brief, 8 µl of hemolyzed blood was incubated in a phosphate buffer solution (100 mM, pH7.5) containing EDTA (0.6 mM), oxidized GSH (3 mM), NADPH (0.25 mM), GSH reductase (0.84 U ml^−1^; Sigma-Aldrich, St. Louis, MO, USA) and Tert-butyl hydroperoxide (0.8 mM; Sigma-Aldrich). The GPx activity was determined as a function of the decrease in NADPH measured at 340 nm and normalized to hemoglobin content (for blood).

The GSH content was measured in 45 µL of whole blood and normalized to blood volume. GSH levels were quantified by a colorimetric approach using a diagnostic kit (Glutathione Assay kit, Calbiochem, San Diego, CA, USA).^57^

### Statistical analysis

Statistical analyses were performed with SPSS (SPSS, Chicago, IL, USA). Differences between patients and controls in handedness, gender, and smoking and cannabis status (users versus non users) were assessed with *Χ*^2^-test. Differences in age and years of parental education were assessed with *t*-test. Group differences in GPx activity and GSH levels were tested with Mann–Whitney *U*-test because of skewness of the distribution of the control group. Outcome measures for brain metrics were hippocampus volume and gFA in the fornix. Group differences in fornix gFA and hippocampus volume were tested with analysis of covariance, with group (patients versus controls) as a between-subject factor and hemisphere as a within-subject factor, and gender and age as covariates. Assumptions of homogeneity of variance between groups were checked through Levene's test. Our sample size was comparable to similar studies and power estimation indicated that it was sufficient to detect effects in the medium range according to Cohen. Correlation analyses were tested with Pearson’s correlation coefficient. Pearson partial correlations were used when correcting for lifestyle factors (BMI, consumption of cigarettes and cannabis) and CPZ equivalents. Correlating brain metrics (hippocampus volume and gFA in the fornix) with redox markers (GSH levels and GPx activity) generated four comparisons, and thus the alpha level to detect significant correlations was set to 0.01. For intra-rater reliability regarding the fornix, six subjects randomly selected were replicated two times by the investigator. Intra-rater correlations for the fornix reached 0.99 for left and right fornix, indicating excellent reliability.

## Results

### Subject characteristics

There were no statistical differences in age, gender, handedness or parental education between the EP and healthy control groups (Table 1), indicating that patients and controls were well matched for these criteria. There was no significant difference in BMI between patients and controls. However there were more smokers and cannabis users in the patient group. Of the total patients, 69% were not using cannabis, whereas 16.7% were mild users and 14.3% were moderate users. At 18 months of follow-up in the TIPP program, diagnostic repartition was as follows: 57% schizophrenia (*n=*24), 16.7% brief psychotic episode (*n=*7), 11.9% schizoaffective disorder (*n=*5), 4.8% bipolar disorder (*n=*2), 4.8% major depression with psychotic features (*n=*2), 2.4% schizophreniform disorder (*n=*1) and 2.4% psychosis not otherwise specified (*n=*1). At the time of this study, 39 of the 42 patients were on antipsychotic medication with an average medication of 340.6±223.6 mg CPZ equivalents (Table 1). The mean duration of illness was 340.6 days (s.d.± 223.6).

### MRI findings

#### Alteration of fornix and hippocampus metrics in EP patients

**Figure 2:**
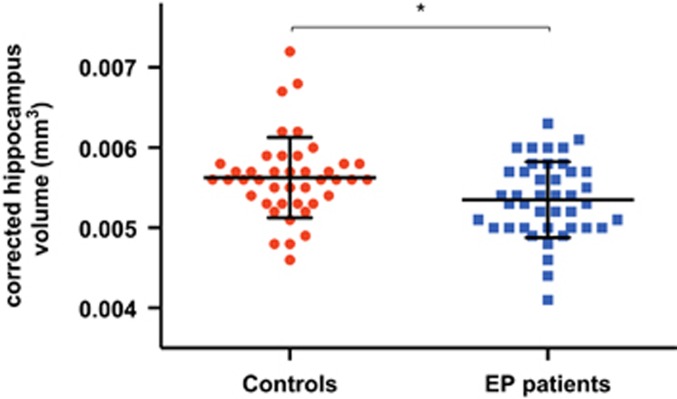
The mean corrected hippocampus volume for controls and early-psychosis (EP) patients. *P<0.05. Error bars denote±s.d. for each group.

Tests of between subjects effects revealed a significant group difference in corrected HV (HV / ICV) with EP subjects exhibiting smaller volume than healthy controls (F(1,80)=6.79, *P=*0.011; Figure 2). There was no main hemisphere effect but a group by hemisphere interaction (F(1,80)=5.98, *P=*0.017) indicating that differences between groups were larger in the left than in the right hemisphere. Left hippocampus in patients were smaller than that in controls (*P=*0.002), whereas right hippocampus difference did not reach statistical significance.

The same analysis of covariance model but comparing absolute hippocampus volume (that is, not normalized by ICV) also revealed significant differences between hemispheres (F(1,80)=12.352, *P=*0.001); both left and right hippocampi were significantly smaller in EP patients than in controls (*P=*0.0002 and *P=*0.005).

**Figure 3:**
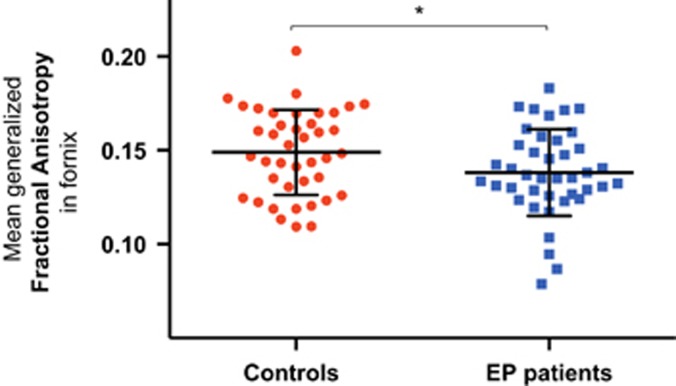
The mean generalized fractional anisotropy (gFA) in the fornix for controls and early-psychosis (EP) patients. *P<0.05. Error bars denote ±s.d. for each group.

Analysis of covariance of fornix gFA showed a significant group effect (F(1,78)=4.482, *P=*0.037) with lower gFA in EP patients than in controls (Figure 3). There was neither hemisphere effect nor group by hemisphere interaction.

There were no significant correlations between CPZ equivalents, number of daily cigarettes, cannabis use (assessed by CMRS) and fornix gFA or absolute hippocampal volume.

#### Correlation between hippocampus and fornix metrics

We used correlation analyses to test the interdependence of fornix and hippocampus integrity. Overall, gFA in the fornix correlated with absolute hippocampus volume (*r=*0.287; *P=*0.009). When groups were studied separately, in controls gFA in the fornix did not correlate with hippocampus volume (*r*=0.086; *P=*0.593). However, these two metrics correlated in patients (*r*=0.388; *P=*0.012) even when corrected for CPZ equivalents and lifestyle factors (cigarette smoking, cannabis and BMI; *r*=0.387; *P=*0.018).

### Correlation between hippocampus and fornix integrity and peripheral redox markers

There were no significant differences between patients and controls in blood GPx activities or GSH levels (Table 1; Supplementary Figure 1).

**Figure 4:**
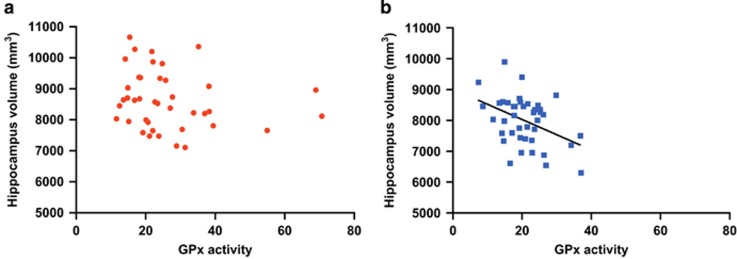
Correlations of hippocampal volume (mm^3^) and glutathione peroxidase (GPx) activity (µmol of GSH/min/g of hemoglobin) in (**a**) controls (*n=*40) and (**b**) early-psychosis (EP) patients (*n=*41). Note that in patients larger hippocampal volume is associated with lower GPx activity (tested with Pearson’s correlation). This relationship is absent in controls. Removing the three outliers with high GPx values (>50) in the control group did not change our conclusions. GSH, glutathione.

In order to test the relationship between fornix and hippocampus structures and GSH-related markers, we correlated imaging metrics with GPx activity and GSH levels. In patients but not in controls, smaller hippocampus volume was associated with higher GPx activity (*r*=0.412; *P=*0.007; Figure 4). In patients, when corrected for medication (CPZ equivalents) and lifestyle factors (cigarette smoking, cannabis and BMI), correlation between hippocampus volume and GPx activity remained significant (*r*=−0.415; *P=*0.012). GPx activity did not correlate with gFA in patients or controls. GSH did not correlate with any brain metrics in patients or controls.

## Discussion

We characterized fornix and hippocampus integrity in EP patients and observed altered diffusion and volumetric properties in the fornix and hippocampus, respectively. We applied for the first time in EP a DSI sequence characterized by strong diffusion weighting. We also provided new evidence that loss of integrity of this circuit is associated with an increased peripheral oxidative status. This observation involves the fornix–hippocampus circuit early in the course of psychosis and, although the detected associations do not imply causality, we provide a plausible hypothesis of the negative impact of oxidative stress on the fornix–hippocampus circuit.

This study was initiated in a reverse translational approach, from GSH-deficient mouse model to EP patients, following the observations of specific structural and functional alterations in the hippocampus^39^ and fornix^36^ in *Gclm*-KO mice.

We used volumetric and diffusion MRI to study the integrity of the fornix–hippocampus circuit.

The DSI sequence, characterized by multiple *b*-values along several diffusion directions, is more sensitive to white matter slow diffusion compartment^59, 60, 61, 62^ (that is, intra-axonal diffusion) than classical diffusion tensor imaging and was only recently applied to schizophrenia.^63^ Patients exhibited decreased gFA in the fornix, which is consistent with two recent studies in first-episode patients.^42^^,^^43^ This is so far the largest cohort showing impaired fornix integrity in the early phase of psychosis. Loss of integrity in the fornix in schizophrenia is a robust finding,^4, 18, 19, 20, 21, 22, 64^ although with mixed results in childhood and adolescent schizophrenia.^65, 66^ Fornix integrity deficit parallels the report of loss of HV in chronic schizophrenia, which is already present in the early phase of psychosis.^8^ In our study, the hippocampus of patients also exhibited volume loss, especially on the left side, which is in line with the findings showing smaller left than right hippocampus in patients^8^ and bilateral involvement only in patients with established schizophrenia.^9^ Finally, volume loss in the hippocampus correlated with loss in integrity in the fornix (gFA), extending findings from chronic schizophrenia to EP.^22, 67^ Taken together, these results indicate that microstructural abnormalities in the fornix are present in the early phase of psychosis and are associated with abnormalities in the hippocampus. Given its early involvement in the disease process, fornix pathology may be an early marker of disease rather than a consequence of chronicity.

There were more cigarette smokers and cannabis users among patients, which may bias our findings. We found however no significant correlations between cigarette smoking or cannabis use and brain metrics in EP patients. Further, in the current sample, more than 2/3 of the sample was not using cannabis and there were no severe or extremely severe cannabis users.

There was no difference between patients and controls in the mean GPx activity, which is in line with a recent meta-analysis by Flatow *et al.*,^31^ showing no change in GPx activity in the early phase, but a decrease in GPx in chronic inpatients and during acute relapse.^31^ In the EP cohort of the current study, we showed for the first time that small HV was associated with high GPx activity (even when controlling for medication and lifestyle factors such as smoking, cannabis and BMI). However, although there was a loss of integrity in the fornix in patients, gFA in the fornix did not correlate with GPx enzymatic activities. This is an unexpected finding, which deserves further consideration. The *gclm-*KO mice exhibit a decrease of 70% in brain GSH levels, which alters the hippocampus^29^ as well as the fornix^36^ integrity and function. It is worthwhile mentioning that this animal model may represent an extreme condition in terms of oxidative stress compared with EP patients. We can thus hypothesize that the hippocampus may be more sensitive to oxidative stress than the fornix bundle, which may partly explain the lack of correlation.

Recent work from our group^68, 69^ showed that in male healthy controls brain GSH levels (as assessed by magnetic resonance spectroscopy) were positively correlated with blood GPx activity, whereas in male patients this correlation was negative. This is coherent with the idea that high blood GPx activity is associated with low brain GSH levels in patients and that high GPx activity reflects a high central oxidative state.

It is well established that oxidative stress and reactive oxygen species (ROS) generation (probably from genetic and environmental risks) lead on one hand to GPx activation and on the other to the activation of the transcription factor NRF2, which in turn upregulate the antioxidant defense system, including GPx.^70, 71^

On the basis of our findings as well as the review by Flatow *et al.*,^31^ it is possible that in the early phase of the disease GPx activity may still be upregulated in response to oxidative stress. In contrast, in the chronic phase this adaptative response is impaired, leading to a vicious circle, as inactivation of GPx causes additional oxidative stress.^72^ This view is compatible with the fact that chronic patients with lower blood-cell GPx activity have higher brain atrophy (measured by computed tomography).^73^

Taken together, these findings indicate that in patients blood-cell GPx activity may represent a potential surrogate marker of the central redox status at least in the early phase of the disease. Given the absence of differences in GPx activity between patients and controls, our findings may be especially relevant to a subgroup of patients with excess of ROS (for example, from environmental impacts) or/and with antioxidant defense deficiency (for example, ‘high-risk’ polymorphism of GCLC, the gene coding for the catalytic subunit of glutamate cysteine ligase).

It is plausible that redox dysregulation/oxidative stress may affect the integrity of the hippocampus, as observed in animal models.^39^ Hippocampal atrophy in schizophrenia has been linked to neuronal atrophy and loss of neuropil,^74^ which may be a consequence of redox dysregulation, although other factors such as inflammation and hypothalamic–pituitary–adrenal axis dysfunction have also been shown to be important for hippocampus integrity.^75^ Much more remains to be learned to fully appreciate the relevance and significance of blood GPx activity along the various stages of the disease. The fact that low level of selenium is a risk factor for schizophrenia^76^ supports the need for further studies on GPx in patients. Indeed, GPx are selenium-containing enzymes whose activities depend on this essential micronutrient.^41, 76^

GPx activity did not correlate with hippocampus volume in healthy controls, a finding that deserves further comments. It is plausible that GPx activity is not a limiting factor as antioxidant defense in the healthy individual as this system involves many factors others than GPx (including superoxide dismutase, catalase, thioredoxin, sulforedoxin and so on). In patients, however, the correlation suggests that this system may become critically limiting.^30^ Blood GSH concentration was not different between patients and controls. We found no association between peripheral GSH levels and hippocampus volume.

Given the lack of correlation of fornix integrity with GPx enzymatic activities and its association with hippocampus volumetric reductions, it may be argued that the hippocampus pathology is primary and loss of fornix integrity a functional consequence of hippocampal atrophy.^22^ Indeed, some interdependence between fornix and hippocampus is expected as the main output of the hippocampus, that is, CA1 and subiculum, conveys fibers via the fimbria and the fornix to the mammillary body.^1^ Further, Schobel *et al.*^13^ showed that ‘at-risk patients’ exhibited hypermetabolism beginning in CA1 and spreading to subiculum after psychosis onset. In the same study, hippocampal atrophy appeared during transition to psychosis. In this regard, it is interesting to note that the fornix connects the hippocampus to the mammillary bodies, which are both structures exhibiting decrease in PV interneurons typically vulnerable to oxidative stress.^15, 77^ However, others have argued that CA1 is relatively spared in schizophrenia at the neuropathological level compared with other subfields,^74^ and hippocampal atrophy may thus not fully explain fornix alterations. Further, fornix pathology is involved early in schizophrenia,^42, 43^ although it is not known whether it is already affected in ‘at-risk patients’. Further investigations will be necessary to evaluate whether and to which extent redox imbalance affects directly fornix integrity in the early phase of psychosis.

Some limitations in our study must be taken into consideration. First, regarding antioxidant defense mechanisms, we assessed GPx and GSH, although other enzymatic and non-enzymatic antioxidants may be important as well. Second, we did not measure ROS, which are reactive and unstable molecules. Sources of ROS are multiple and include known environmental risk factors for schizophrenia but also genetic factors, dopamine metabolism, antipsychotics and inflammation.^30, 78^ In addition, mitochondria electron transport chain leakage is an important source of ROS and previous work indicated mitochondrial impairment in schizophrenia.^78, 79, 80, 81^ Oxidation and peroxidation of macromolecules (lipids, proteins and DNA; that is, consequence of excessive oxidative stress) have also been repeatedly reported in patients^30, 31, 82^ and may constitute an alternative to ROS assessment. Some additional caveats must also be considered. We found a decrease in gFA, a metric that can be modulated by myelin integrity but also other factors such as axonal size and volume of water surrounding axons,^83^ and we can only speculate about which factor is the most implicated. Second, our patient sample was not neuroleptic-naive. However, we controlled for CPZ equivalents, which did not correlate with gFA or HV in patients. Further, our sample is in the early phase of psychosis and was not exposed to chronic antipsychotic treatment. It is thus unlikely that our findings are fully explained by antipsychotic medication.

## Conclusion

Our study shows that GPx activity is a peripheral correlate of hippocampus integrity in EP patients, which suggests a role for oxidative stress in the disease mechanism. Further translational research is needed to determine to which degree peripheral markers reflect central mechanisms in order to establish clinically useful peripheral biomarkers. In addition, the identification of oxidative stress as a potential contributing mechanism to psychosis suggests its implication as a mediating factor between environmental stressors (such as trauma, for example, Alameda *et al.*^84^) and development of such disorders. These possible mechanisms should be explored in future work. Finally, the investigation of fornix–hippocampus integrity in the prodromal phase of psychosis is warranted, considering that it may be useful as an early marker of risk.

## Figures and Tables

**Table 1:** Subjects' characteristics

	*Early psychosis patients (*N*=42)*	*Control subjects (*N*=42)*	P*-value*
Age, mean±s.d.	25.0±5.4	25.3±5.3	NS^a^
Gender, M/F	28/14	29/13	NS^b^
Handedness right/left/ambidextrous	36/4/2	35/6/1	NS^b^
Education of parents (years)	13.2±4.1	14.1±4.7	NS^a^
GPx activity (µmol/min/g of Hb)^c^	20.8±6.6^f^	26.4±13.6^e^	NS^d^
GSH (µmol/ml)^c^	0.79±0.3	0.69±0.3	NS^d^
Cigarettes users/non-user	24/18	1/36	*P<*0.05^b^
Cannabis user/non-user	13/29	1/38	*P<*0.05^b^
BMI, mean±s.d.	23.7	22.5^e^	NS^a^
Duration of illness, days	630.7±425.9	—	—
CPZ eq., mean±s.d.	340.6±223.6	—	—
PANSS positive, mean±s.d.	13.6±4.6	—	—
PANSS negative, mean±s.d.	15.6±5.7	—	—
PANSS general, mean±s.d.	33.6±8.9	—	—

Abbreviations: Age in years; BMI, body mass index (kg/m^2^); CPZ eq., antipsychotic medication converted to chlorpromazine equivalent, in mg; GPx, glutathione peroxidase; GSH, glutathione; Hb, hemoglobin; NS, nonsignificant; PANSS, Positive and Negative Syndrome Scale:

positive symptom score, negative symptom score, general symptom score.

a *t*-test.

b *Χ*^2^-test.

c GPx and GSH measurements were conducted on blood and normalized, respectively, to Hb and blood volume.

d Mann–Whitney *U*-test.

e Data missing for two subjects.

f Data missing for one subject.
