# Supplementary material for: Impaired fornix–hippocampus integrity is linked to peripheral glutathione peroxidase in early psychosis
Source: Transl Psychiatry. 2016 Jul 26;6(7):e859–. doi: 10.1038/tp.2016.117 (PMC5545707; doi:10.1038/tp.2016.117)
Supplement: Supplementary Figure 1 [file tp2016117x1.ppt]

## Slide 1
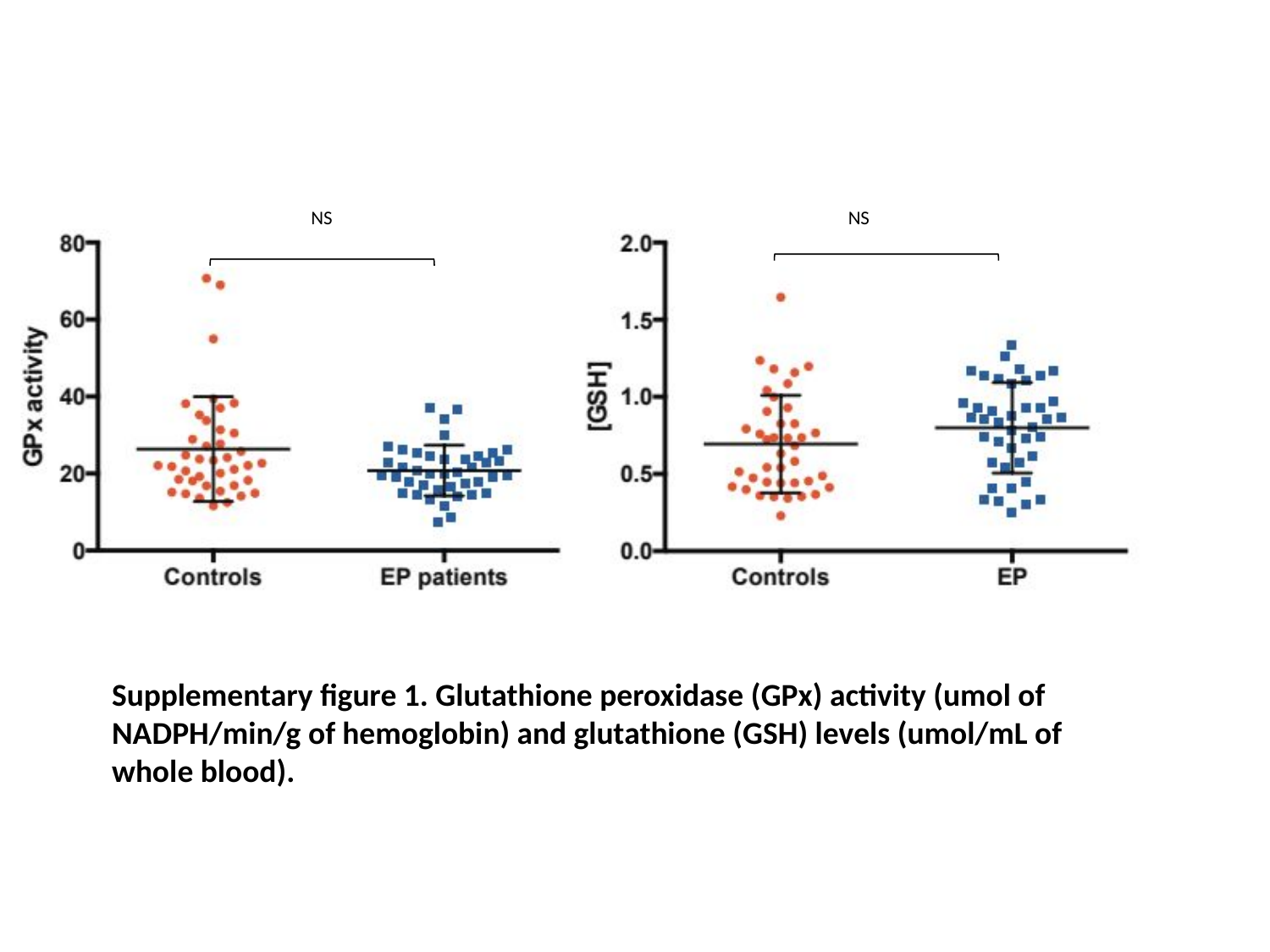

NS
NS
Supplementary figure 1. Glutathione peroxidase (GPx) activity (umol of NADPH/min/g of hemoglobin) and glutathione (GSH) levels (umol/mL of whole blood).
